# Evaluation of optimal monoenergetic images acquired by dual-energy CT in the diagnosis of T staging of thoracic esophageal cancer

**DOI:** 10.1186/s13244-023-01381-1

**Published:** 2023-02-10

**Authors:** Fanrong Cheng, Yan Liu, Lihong Du, Lei Wang, Lan Li, Jinfang Shi, Xiaoxia Wang, Jiuquan Zhang

**Affiliations:** 1 Department of Radiology, Chongqing Key Laboratory for Intelligent Oncology in Breast Cancer (iCQBC), Chongqing University Cancer Hospital, Chongqing, 400030 China; 2 People’s Hospital of Rongchang District, Chongqing, 402460 China

**Keywords:** Dual-energy computed tomography, Monoenergetic images, Polyenergetic images, Esophageal cancer, Quantitative parameters

## Abstract

**Objectives:**

The purpose of our study was to objectively and subjectively assess optimal monoenergetic image (MEI (+)) characteristics from dual-energy CT (DECT) and the diagnostic performance for the T staging in patients with thoracic esophageal cancer (EC).

**Methods:**

In this retrospective study, patients with histopathologically confirmed EC who underwent DECT from September 2019 to December 2020 were enrolled. One standard polyenergetic image (PEI) and five MEI (+) were reconstructed. Two readers independently assessed the lesion conspicuity subjectively and calculated the contrast-to-noise ratio (CNR) and the signal-to-noise ratio (SNR) of EC. Two readers independently assessed the T stage on the optimal MEI (+) and PEI subjectively. Multiple quantitative parameters were measured to assess the diagnostic performance to identify T1-2 from T3-4 in EC patients.

**Results:**

The study included 68 patients. Subjectively, primary tumor delineation received the highest ratings in MEI (+) _40 keV_ of the venous phase. Objectively, MEI (+) images showed significantly higher SNR compared with PEI (*p* < 0.05), peaking at MEI (+) _40 keV_ in the venous phase. CNR of tumor (MEI (+) _40 keV -80 keV_) was all significantly higher than PEI in arterial and venous phases (*p* < 0.05), peaking at MEI (+) _40 keV_ in venous phases. The agreement between MEI (+) _40 keV_ and pathologic T categories was 81.63% (40/49). Rho values in venous phases had excellent diagnostic efficiency for identifying T1-2 from T3-4 (AUC = 0.84).

**Conclusions:**

MEI (+) reconstructions at low keV in the venous phase improved the assessment of lesion conspicuity and also have great potential for preoperative assessment of T staging in patients with EC.

## Key points

- Precise preoperative T staging in esophageal cancer patients is critical.
- Dual-energy CT is widely used to improve the SNR and CNR of images.
- The multiple quantitative parameters from DECT exhibited potential in distinguishing T staging.

## Introduction

Esophageal cancer (EC) is the most common malignant tumor of the digestive tract, which ranks seventh in terms of incidence (604,000 new cases) and sixth in mortality overall (544,000 deaths) in 2020 global cancer statistics [1]. Surgery remains the first-line treatment for early stage (T1 and T2), while neoadjuvant chemotherapy (NAC) followed by esophagectomy is recommended for locally advanced stage (T3 and T4a) in patients with EC [2]. In addition, according to the National Comprehensive Cancer Network (NCCN) guideline, additional preoperative chemoradiation or perioperative chemotherapy is used to improve survival for locally advanced resectable EC patients [3]. Therefore, precise preoperative T staging in EC patients is critical in determining operation and treatment options [4]. In addition, as the thoracic esophagus is adjacent to the heart and aorta, the motion artifacts are significantly more obvious than the cervical esophagus and esophagogastric junction, making T staging more difficult.

Endoscopic ultrasound (EUS) has been deemed to a standard method for preoperative evaluation of T staging in patients with EC [5]. However, the presence of tumoral obstruction in the esophageal lumen can prevent the progression of the EUS in up to 30% of the cases, making the value of the EUS limited [6, 7]. Meanwhile, due to the high soft tissue resolution in MRI, a recent study displayed the high sensitivity (98%) and accuracy (96%) of MRI for T staging in patients with EC [4]. Unfortunately, due to the posterior location of the esophagus in the mediastinum, motion artifacts resulting from breathing, heartbeat, swallowing, peristalsis, and magnetic susceptibility artifacts limited MRI routine application in clinical practice.

According to the guidelines of the American Joint Committee on Cancer (AJCC), computer tomography (CT) is the primary recommendation and commonly used noninvasive technique in the preoperative evaluation of T staging in patients with EC. However, conventional CT cannot accurately show the boundary of tumor and is difficult to differentiate the primary lesion and the surrounding tissues due to the low contrast-to-noise ratio (CNR) and the signal-to-noise ratio (SNR). As a result, the early tumor stages (T1 and T2) were hard to be reliably differentiated, resulting in a detection rate of only 30% for T1 stage tumors [8].

Recently, the noise-optimized virtual monoenergetic images (MEI (+)) derived from dual-energy CT (DECT) are widely used to improve the SNR and CNR, which was superior tumor visibility to polyenergetic images (PEI) [9–11]. Zopfs et al. demonstrated that virtual monoenergetic images at 40–60 keV improve qualitative assessment of the EC lesion and depiction of lymph nodes and vessels at pretherapeutic [12], while this study was based on adenocarcinoma of the esophagogastric junction and did not explore the clinical diagnostic value of DECT in preoperative T staging of EC. On the other hand, DECT can provide multiple quantitative information about tissue composition, overcoming the limitations of attenuation-based conventional single-energy CT imaging [13]. A previous study indicates an added value of DECT-derived MEI (+) and iodine density (ID) maps in T staging of colorectal cancer; the overall accuracy was 90.3% [14].

Therefore, our aim was to assess the tumor visualization on MEI (+) and PEI objectively and subjectively and investigate the diagnostic performance of subjective assessment combined with multiple quantitative parameters acquired from DECT for evaluation of T staging in patients with thoracic EC.

## Materials and methods

### Patient inclusion

**Fig. 1:**
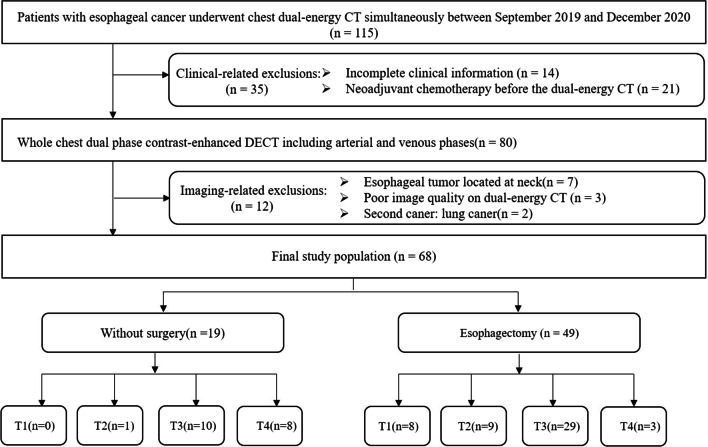
Flowchart showing overview of patients inclusion and exclusion

This retrospective study was approved by the ethics committee of our hospital. The need for written informed consent was waived. All consecutive patients who had chest DECT and endoscopy were recruited from September 2019 to December 2020. Inclusion criteria were as follows: (i) single lesions located in the thoracic portion; (ii) all patients diagnosed with EC by endoscopy and biopsy; and (iii) contrast-enhanced ultrasound including arterial phase and venous phase DECT of the chest. Exclusion criteria were as follows: (i) clinical data missing or incomplete; (ii) second tumor besides EC; (iii) radiotherapy or chemotherapy treatment before DECT; and (iv) poor image quality on DECT. After applying these inclusion and exclusion criteria, 68 patients with EC were analyzed for tumor visualization on MEI (+) and PEI objectively and subjectively; of these 49 patients received radical surgery and obtained pathologically confirmed T staging. The workflow chart is shown in Fig. 1.

Patient clinicopathological data were collected, which included age, sex, tumor location, tumor histology, pathological grading, clinical TN staging, and pathological TN staging. The clinical T and N staging was established by DECT. T staging was performed according to the International Union Against Cancer/American Joint Committee on Cancer (UICC/AJCC) TNM classification for EC (7th edition, 2011), details as follows: T1-tumor invades lamina propria, muscularis mucosae or submucosa; T2-tumor invades muscularis propria but without breaking through muscularis propria; T3-tumor invades adventitia; T4a-tumor invades pleura, pericardium, azygos vein, diaphragm or peritoneum; and T4b-tumor invades other adjacent structures, such as the aorta, vertebral body, and trachea.

### DECT image acquisition

All patients were scanned using a 64-detector CT scanner (SOMATOM Drive, Siemens Healthineers) in dual-energy mode through two X-ray tubes with different kV tube voltages (tube A, 100 kV; tube B, Sn 140 kV), using a tin filter for the high-voltage tube. Automatic exposure control (CARE Dose 4D, Siemens Healthineers) was used in all scans. The parameters of scanners were as follows: collimation, 64 × 0.6 mm; rotation time, 0.28 s; pitch, 0.55; reference tube current time product, 71 mAs for the 100 kV tube and 60 mAs for the Sn140 kV tube; reformatted section thickness, 1.5 mm; reformatted section increment, 1.5 mm.

First, all patients were scanned non-contrast DECT images of chest. Then, acquired contrast-enhanced images and iodinated nonionic contrast media (ioversol, Hengrui Medicine) were administered through the ulnar vein at a dose of 1.5 mL/kg with a flow rate of 2.5 mL/s, followed by a bolus injection of 30 mL of saline at the same flow rate. The arterial phase was acquired after the injections 10 s (average, 35 ± 5 s). The scan delay time for the venous phase scanning was 25 s after the end of the arterial phase scanning (average, 60 ± 7 s).

### DECT image reconstruction

Reconstructed DECT image data were post-processed on syngo.via workstation (VB20A, Dual Energy, Siemens Healthineers). The MEI (+) images were reconstructed at 40, 50, 60, 70, and 80 keV levels, and the PEI was reconstructed by applying the blending factor of 0.4 (M_0.4; 40% of the low kV and 60% of the high kV spectrum).

### Subjective image analysis

The images were analyzed on MEI (+) images (40–80 keV) and PEI independently by two radiologists with 3 and 5 years of chest CT experience, respectively. Two readers individually rated each image series regarding the following categories using 5-point Likert scale: (i) image sharpness (ranging from 1 = distinct blurring to 5 = no apparent blurring); (ii) image noise (defined as image graininess: ranging from 1 = extensive image noise to 5 = no apparent noise); (iii) lesion margin (demarcation of lesion margins: ranging from 1 = no visual demarcation to 5 = perfect demarcation of contours); and (iv) lesion inside (the definition of cystic necrosis inside the lesion: 1 = nondiagnostic, 2 = poor, 3 = sufficient, 4 = good, 5 = excellent).

### Objective image analysis

Objective image analysis was also performed on five sets of MEI (+) and PEI. The first region of interest (ROI) was located in the primary EC at the maximum diameter without areas of apparent cystic necrosis, blood vessel and air. The second ROI was located in the normal esophageal wall; and the last ROI was located background of the air. The average size of the three ROIs was 5–10 mm^2^. The position and size of the ROIs were kept constant in all sets of MEI (+) and PEI in both the arterial and venous phases. The mean attenuation (Hu) of EC lesion and normal esophageal wall and the standard deviation (SD) of the air were recorded; then, the SNR and CNR of EC lesions were calculated according to the following formulas:

$${\text{CNR }} = {\text{Attenuation}}_{{({\text{EC}})}} - {\text{Attenuation}}_{{({\text{normal esophageal wall}})}} /{\text{SD}}_{{({\text{air}})}}$$

$${\text{SNR }} = {\text{Attenuation}}_{{\left( {{\text{EC}}} \right)}} /{\text{SD}}_{{\left( {{\text{air}}} \right)}}$$

### Subjective evaluation of T staging

Subjective evaluation of T staging was performed in EC patients who received radical surgery. Two radiologists with 5 and 3 years of experience, who were blinded to the histopathological data, reviewed the optimal MEI (+) and the PEI to evaluate the T staging according to the UICC/AJCC TNM classification for EC (7th edition, 2011) independently. When the two reader's assessment of T staging appears inconsistent, they would discuss to achieve a consensus result.

### Multiparameter differential T staging

Multiple quantitative parameters were measured to differential T1–2 from T3–4 staging, including: (i) the effective atomic number (Z_eff_) of non-contrast image; (ii) the attenuation (Hu) in arterial and venous phases of the optimal MEI (+); (iii) the normalized iodine concentration (NIC) obtained by iodine concentration of lesions that divided the iodine concentration of the aorta in arterial and venous phases; and (iv) electron density (Rho) in arterial and venous phases.

### Statistical analysis

The statistical analyses were performed using software (IBM SPSS software, version 23). The data distribution was assessed using the Kolmogorov–Smirnov test. Subjective Likert scores and CNR and SNR were compared using the Wilcoxon test with adjustment for multiple comparisons, where applicable. The Kappa concordance test was used to evaluate the interobserver agreement of subjective Likert scores; and a kappa value ≤ 0.20 indicates poor agreement, 0.21–0.40 is fair, 0.41–0.60 is moderate, 0.61–0.80 is good, and 0.81–1.00 is excellent. The agreement of T staging between MEI (+) and PEI with those assigned after postoperative histopathologic examination was calculated. For all multiple quantitative parameters analysis to identify T1-2 and the T3-4 in EC patients, the area under the receiver operating characteristic (ROC) curve (AUC), sensitivity, specificity, positive predictive value (PPV), negative predictive value (NPV), and accuracy were calculated at maximal Youden’s index. The level of significance was set at *p* ≤ 0.05.

## Results

### Patients

A total of 68 patients including 52 men (67.37 ± 6.53 years, 52–85 years) and 16 women (67.50 ± 12.52 years, 51–85 years) were enrolled in our study. Of these, 49/68 patients received radical surgery after a DECT scan (within a week) and obtained pathologically confirmed T and N staging, while 19/68 patients were treated by systemic therapy: (i) chemotherapy (*n* = 9); (ii) radiotherapy (*n* = 4); (iii) concurrent chemoradiotherapy (*n* = 3); and (iv) traditional Chinese medicine (*n* = 3). In addition, 49 patients received radical surgery, and the pathological T staging was as follows: 7 (14.3%) patients with T1, 10 (20.4%) patients with T2, 29 (59.2%) patients with T3, and 3 (6.1%) patients with T4, while 19 patients received systemic therapy, and the clinical T staging is as follows: 1 (5.2%) patient with T2, 10 (52.6%) patients with T3, and 8 (42.1%) patients with T4. Volume computed tomography dose index (CTDIvol) and dose length product (DLP) for every patient in each phase scanning of chest examination was estimated to 3.92 ± 1.40mGY and 123.11 ± 60.73 mGy*cm. The detail of patient clinicopathological data can be seen in Table 1.

**Table 1:** Patient clinicopathological data of esophageal cancer patients

Characteristic	Number
Age, mean ± SD, years (range)	67.4 ± 8.22 (51–85)
*Sex* (%)	
Female	52 (76.5)
Male	16 (23.5)
*Tumor location of the thoracic* (%)	
Upper	7 (10.3)
Middle	49 (72.1)
Lower	12 (17.6)
*Tumor histopathology* (%)	
Squamous cell carcinoma	66 (97)
Other	2 (3)
Tumor grading (%)	
Well differentiated	21 (30.8)
Moderately differentiated	34 (50)
Poorly differentiated	13 (19.1)
*Systemic therapy group (n*** = ***19)*	
*cT stage* (%)	
T2	1 (5.2)
T3	10 (52.6)
T4	8 (42.1)
cN stage (%)	
N0	3 (15.8)
N1	4 (21.1)
N2	12 (63.1)
*Radical surgery group (n*** = ***49)*	
*pT stage* (%)	
T1	8 (16.3)
T2	9 (18.4)
T3	29 (59.2)
T4	3 (6.1)
*pN stage* (%)	
N0	28 (57.1)
N1	13 (26.5)
N2	6 (12.2)
N3	2 (4.1)

### Subjective image analysis

**Fig. 2:**
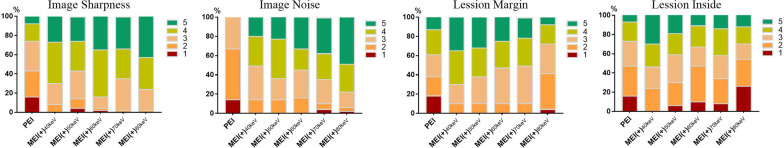
Results of the subjective assessment. Image sharpness received significantly higher ratings in MEI (+) 40–80 keV compared to PEI. For image noise, the highest scores were assigned in MEI (+) 80 keV. Diagnostic certainty regarding lesion margin was optimal in MEI (+) 40 keV, while assessment of the lesion inside received the highest score in MEI (+) 40 keV

Inter-reader agreement was excellent for subjective image analysis in the arterial phase (*k* = 0.95 for image sharpness, *k* = 0.97 for image noise, *k* = 0.95 for lesion margin, *k* = 0.96 for lesion inside) and was good in the venous phase (*k* = 0.74 for image sharpness, *k* = 0.75 for image noise, *k* = 0.71 for lesion margin, *k* = 0.73 for lesion inside). Table 2 and Fig. 2 show the results of the Likert scores on PEI and MEI (+)_40–80 keV_ in arterial and venous phases, respectively. Reader assigned highest scores to MEI (+) _40 keV_ in the venous phase for delineation of lesion margin and lesion inside (all *p* < 0.01), and lower scores for delineation of image sharpness (*p* < 0.01). Meanwhile, regarding the assessment of the image noise, MEI (+) _40 keV_ attained more evident than both PEI and MEI (+) _60–80 keV_ (*p* < 0.01).

**Table 2:** Results of the Likert scores on PEI and MEI (+) _40 keV-80 keV_ in arterial and venous phases

	Image sharpness	Image noise	Lesion margin	Lesion inside
*Arterial phase*				
PEI	3 (3,4)	3 (3,4)	3 (2.5,3)	3 (2,3)
40 keV	3 (2,3) *	2 (2,3) *	4 (3.5,5) *	4 (4,5) *
50 keV	3 (3,4)	3 (2,3) *	4 (4,5) *	4 (3,4) *
60 keV	4 (3,4)	4 (3,4)	4 (3,4) *	3 (3,4) *
70 keV	4 (4,5) *	4 (4,5) *	3 (3,4) *	3 (2,3)
80 keV	5 (4.5,5) *	5 (4,5) *	3 (3,4) *	2 (2,3)
*Venous phase*				
PEI	3 (3,4)	3 (3,4)	3 (3,3.5)	3 (2,3)
40 keV	3 (2,3) *	2 (1,3) *	5 (4,5) *	5 (5,5) *
50 keV	3 (2.5,4)	3 (2,3)	5 (4,5) *	4 (4,4) *
60 keV	4 (3,5) *	4 (3,5)	3 (2.5,4)	3 (2,4) *
70 keV	4 (3,4) *	4 (3,5) *	3 (3,5) *	3 (2,3)
80 keV	4 (4,5) *	4 (3,5) *	3 (3,4) *	3 (2,4)

* indicate significant differences (*p* < 0.05) of MEI (+) _40–80 keV_ compared to PEI. Likert scores are presented as medians and their interquartile ranges (IQR)

### Objective image analysis

SNR for primary esophageal tumor showed an increasing tendency with decreasing keV levels of MEI (+). MEI (+) _40–80 keV_ showed significantly higher SNR than PEI. The highest SNR value of the primary tumor (12.96 ± 2.92) was found in MEI (+) _50 keV_ in the arterial phase, which was significantly higher than the referring value encountered in PEI (primary tumor: 8.10 ± 1.49) (*p* < 0.05), but there were no significant differences between MEI (+) _50 keV_ and the other MEI (+) (all *p* > 0.05). Concordant to the results found for the arterial phase, SNR in the venous phase in MEI (+) _40–80 keV_ was significantly higher than in PEI showing the largest difference between MEI (+) and PEI (all *p* < 0.05). The highest SNR value of the primary tumor (17.14 ± 4.21) was found in MEI (+) _40 keV_, which was significantly higher than the PEI and MEI (+) _80 keV_ (primary tumor: 9.56 ± 1.91, 14.14 ± 4.23) (*p* < 0.05).

**Fig. 3:**
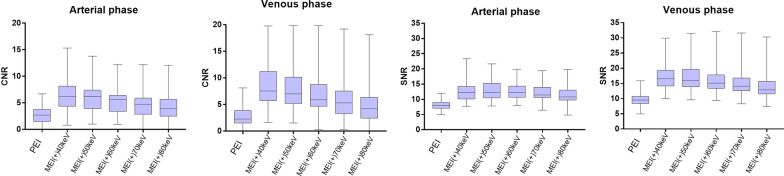
CNR and SNR for esophageal cancer vs. normal esophageal wall in arterial and venous phase. Increasing CNR or SNR values with decreasing energy levels of noise-optimized virtual monoenergetic images can be appreciated. Asterisks indicate statistically significant differences compared to each other. Besides, CNR or SNR values of PEI were significantly lower than MEI (+) 40–80 keV (*p* < 0.05)

CNR of the primary tumor was significantly higher in MEI (+) _40–80 keV_ than in PEI in both arterial and venous phases. The CNR showed the highest value at MEI (+) _40 keV_ ([arterial phase: 6.53 ± 3.04], [venous phase: 8.54 ± 3.82]), and they were significantly higher than the CNR of PEI and of MEI (+) _80 keV_ (adjusted *p* range, < 0.01–0.04). Detailed results of the objective image analysis are shown in Table 3 and Fig. 3.

**Table 3:** Quantitative values of esophageal cancer for SNR and CNR in the arterial and venous phases

	PEI	MEI (+) _40 keV_	MEI (+) _50 keV_	MEI (+) _60 keV_	MEI (+) _70 keV_	MEI (+) _80 keV_	*p*
*Arterial phase*							
SNR	8.10 ± 1.49	12.48 ± 3.02	12.96 ± 2.92	12.73 ± 2.75	12.26 ± 2.76	11.61 ± 2.87	0.01*
CNR	2.69 ± 1.51	6.53 ± 3.04	6.05 ± 2.87	5.43 ± 2.65	4.79 ± 2.59	4.21 ± 2.54	0.01*
*Venous phase*							
SNR	9.56 ± 1.91	17.14 ± 4.21	16.90 ± 4.19	16.11 ± 4.16	15.28 ± 4.26	14.14 ± 4.23	0.01*
CNR	2.91 ± 1.95	8.54 ± 3.82	7.71 ± 3.59	6.56 ± 3.42	5.63 ± 3.38	4.74 ± 3.34	0.01*

*p* < 0.05 indicate statistically significant. *The nonparametric with Kruskal–Wallis H test for non-normally distributed data

*ANOVA* Analysis of variance, *CNR* Contrast-to-noise ratio, *keV* Kiloelectron volt, *MEI* (+) Noise-optimized virtual monoenergetic images, *PEI* Polyenergetic images, *SNR* Signal contrast-to-noise ratio, Esophageal cancer (*EC*)

### Subjective evaluation of T staging

**Fig. 4:**
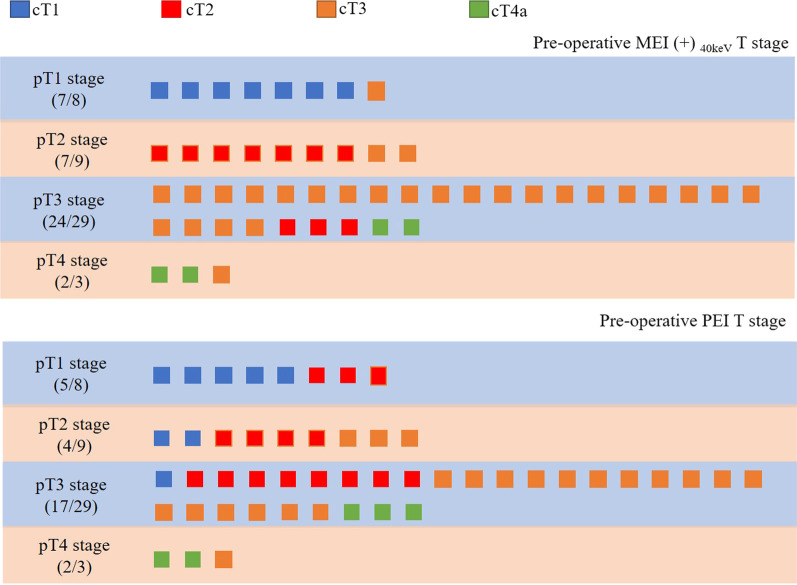
Agreement between MEI (+) 40 keV (or PEI) and pathologic T categories. Agreement between MEI (+) 40 keV and pathologic T categories was 81.63% (40/49). Agreement between PEI and pathologic T categories was 48.97% (24/49)

**Fig. 5:**
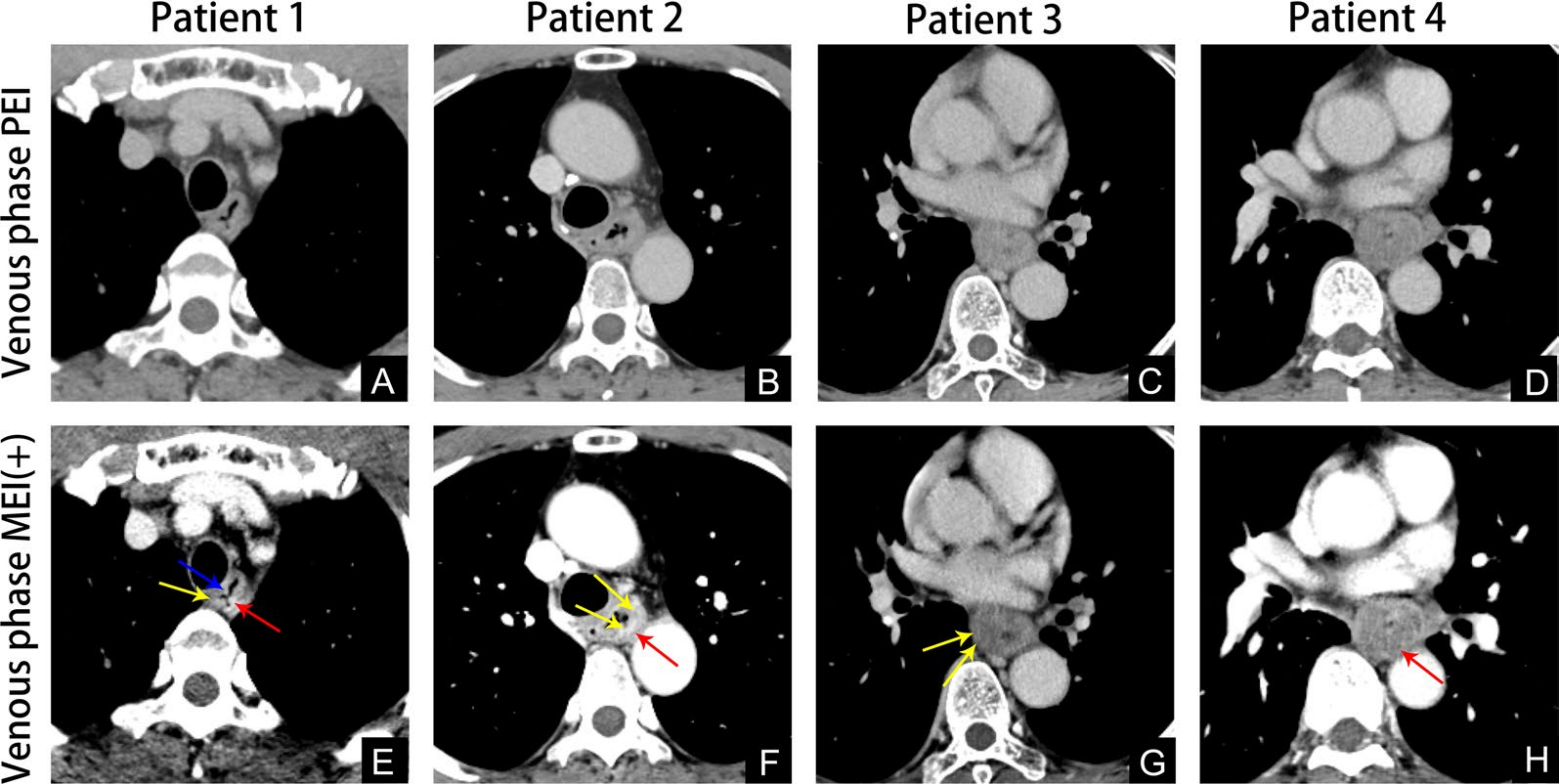
**A** and **E**, 66-year-old man with esophageal cancer, venous phase PEI image **A**, venous phase MEI (+) 40 keV image **E**, PEI image CT cannot distinguish the tumor, MEI (+) 40 keV shows the high-density muscularis mucosae (blue arrow) and muscularis propria (yellow arrow) are interrupted, and the lesion (Red arrow) is staged as T1b. **B** and **F**, 64-year-old man with esophageal cancer, venous phase PEI image **B**, venous phase MEI (+) 40 keV image **F**, PEI image CT cannot distinguish the boundary of tumor, MEI (+) 40 keV shows the tumor-invaded muscularis propria (yellow arrow) and the boundary of lesion is clear (Red arrow). The lesion is staged as T2. **C** and **G**, 69-year-old man with esophageal cancer, venous phase PEI image **C**, venous phase MEI (+) 40 keV image **G**, PEI image CT show the boundary of tumor is not clear, MEI (+) 40 keV shows the tumor-invaded serosa (yellow arrow) and cystic necrosis was evident in the lesion. The lesion is staged as T3. **D** and **H**, 62-year-old man with esophageal cancer, venous phase PEI image **D**, venous phase MEI (+) 40 keV image **H**, PEI and MEI (+) 40 keV image CT all show the tumor-invaded descending aorta (red arrow). The lesion is staged as T4b

Based on the results of the objective and subjective evaluation, we selected the venous phase of 40 keV MEI (+) for evaluation of T staging compared with PEI. The final cT staging was based on the group consensus of the two radiologists. Agreement between MEI (+) _40 keV_ and pathologic T categories was 81.63% (40/49). One stage T1 and two stage T2 patients were overestimated as stage T3, and two T3 patients were overestimated as stage T4, whereas three T3 cases were underestimated to be stage T2 and one T4 patient was underestimated as T3. Agreement between PEI and pathologic T categories was 48.97% (24/49). About 50% of patients were overestimated or underestimated, most errors occur differentiating the T2 stage from T1 and T3 stage and T3 stage from T2 stage lesions. Detailed values are shown in Table 4 and Fig. 4. Figure 5 provides an example of lesions assigned categories T1–T4a.

**Table 4:** MEI (+) _40 keV_, PEI versus postoperative histopathology for T stage of esophageal cancer in the venous phase

Postoperative pathologic T stage	Preoperative MEI (+)_40 keV_ T stage				Preoperative PEI T stage			
	T1	T2	T3	T4	T1	T2	T3	T4
T1 (*n* = 8)	7	0	1	0	5	3	0	0
T2 (*n* = 9)	0	7	2	0	2	4	3	0
T3 (*n* = 29)	0	3	24	2	1	8	17	3
T4 (*n* = 3)	0	0	1	2	0	0	1	2

### Multiparameter differential T staging

The multiple quantitative parameters between the early stage (T1-2) and advanced stage (T3-4) of EC are shown in Table 5. There was no statistically significant difference (*p* > 0.05) in the value of Z_eff_ and CT attenuation values. The Rho of arterial and venous phases was significantly higher in the T3-4 staging group than in the T1-2 staging group (*p* = 0.01 and *p* = 0.00, respectively), while the NIC values of arterial and venous phases were significantly lower in the T3-4 staging group than in the T1-2 staging group (*p* = 0.02 and *p* = 0.01, respectively). ROC curve analysis showed that the Rho of the venous phase had excellent diagnostic efficiency, with an AUC of 0.84, a sensitivity of 91.12%, a specificity of 67.54%, and accuracy of 82.35%

**Table 5:** Distribution of quantitative Z_eff_ of unenhanced image, attenuation, NIC and Rho parameters among different pT staging in arterial and venous phases

Parameter	pT1-2	pT3-4	*p*	AUC (95% CI)	Sensitivity (%) (95% CI)	Specificity (%) (95% CI)	PPV (95% CI)	NPV (95% CI)	Accuracy (%)	Cutoff value
Z_eff_	7.51 ± 0.19	7.49 ± 0.18	0.64	0.51 (0.33 – 0.67)	96.97 (84.2 – 99.9)	22.22 (6.40 – 47.6)	69.60 (54.20 –82.30)	80.00 (28.40 –99.50)	66.67	7.69
Attenuation_(arterial)_	184.06 ± 54.53	171.90 ± 46.63	0.41	0.57 (0.42 –0.71)	83.87 (66.30 –94.5)	50.00 (26.00 –74.00)	74.30 (56.70 –87.50)	64.30 (35.10 –87.20)	71.42	203.63
Attenuation_(venous)_	236.49 ± 40.81	224.23 ± 53.75	0.41	0.61 (0.45 ~ 0.77)	59.38 (40.60 –76.30)	70.59 (44.00 –89.70)	78.30 (56.30 –92.50)	50.00 (29.90 –70.10)	63.26	223.97
NIC _(arterial)_	2.29 ± 1.14	1.75 ± 0.50	0.02*	0.63 (0.45 –0.81)	96.87 (83.80 –99.90)	41.18 (18.40 –67.10)	74.40 (58.80 –86.50)	87.50 (47.30 –99.70)	78.43	2.55
NIC _(venous)_	2.90 (2.67, 3.45)	2.30 (1.9, 2.95)	0.01*	0.72 (0.58 –0.86)	59.38 (40.60 –76.30)	82.35 (56.60 –96.20)	87.00 (66.40 –97.20)	53.60 (33.90 –72.50)	68.62	2.55
Rho_(arterial)_	37.75 (27.45, 45.25)	43.90 (39.8, 47.7)	0.01*	0.72 (0.67 –0.88)	100 (89.40 –100.0)	38.89 (17.30 –64.30)	75.00 (59.70 –86.80)	100.00 (59.00 –100.00)	72.54	41.65
Rho_(venous)_	31.66 ± 8.51	42.23 ± 5.60	0.00*	**0.84 (0.73** –**0.96)**	91.12 (71.00 –96.50)	67.54 (38.30 ~ 85.80)	81.60 (65.70 –92.30)	84.6 (54.60 –98.10)	82.35	35.35

Highest diagnostic effectiveness value is shown in bold

^*^p < 0.05 indicate statistically significant

## Discussion

In this study, we found that MEI (+) _40 keV_ in the venous phase had improved the tumor visualization by objective and subjective analysis and also superior to PEI in assessing T staging. Besides, our findings indicate that multiple quantitative parameters acquired by DECT were useful for the preoperative T staging. Particularly, Rho of the venous phase had the highest diagnostic efficiency to identify T1-2 from T3-4 in EC patients.

In recent years, there were many studies that demonstrated MEI (+) with low keV can improve image quality in chest, abdominal, cerebral, and soft tissue lesions without increasing the radiation dose [15–18]. MEI (+) assist achieved higher CNR and SNR images than conventional CT. The dose in our study was similar to this reported by Yue Zhou et al. [19]. Tilman Hickethiera et al. also verified MEI (+) 40 keV venous-phase chest CT examinations can reduce doses and improve the quality of images [10]. The MEI (+) technique performs recombination based on spatial frequency, which reduces the image noise at lower levels and improves the image contrast at higher energies to obtain the best image contrast [20]; thus, MEI (+) improves the potential of evaluation for T staging [21]. Combined with the high contrast of MEI (+) _40 keV_ and obvious enhancement of the lesion in the venous phase, the depth of lesion involvement to the esophageal wall was subjectively evaluated. The diagnostic accuracy of MEI (+) _40 keV_ in the venous phase evaluation for T staging was satisfactory. Our results were better than previous reports whose accuracy of local staging was 76.3% or 52.50% [19, 22]. Although, in our study, a proportion of patients were overestimated or underestimated between T2 and T3, our results show that the mass margins were significantly clearer and the lesion of mucosal layer is more obvious in MEI (+) _40 keV_ than in PEI. DECT application in EC patients also can clearly depict the addition structure (such as lymph node or vascular) of EC [12].

Dual-energy CT can obtain multiparameter images such as substance separation, virtual single energy, effective atomic number, and energy spectrum curve, which can qualitatively and quantitatively provide more valuable information for the differential diagnosis of lesions, determination of pathological types and aggressiveness of cancers, and prediction of efficacy of neoadjuvant therapy [18, 23–25]. However, the multiparameters of DECT are rarely used for esophageal cancer staging. Recently, there has been a wave of application of magnetic resonance imaging (MRI) in the T staging of EC [26, 27]. MRI, with its high soft tissue resolution, achieves up to 96% accuracy in T staging of EC [26], which is higher than ours. These studies rely on many advanced technologies, such as free-breathing radial VIBE. Currently, these technologies are available only in a few healthcare facilities. So, conventional MRI is still significantly disturbed by motion artifacts and is limited in the detection of EC. Moreover, due to the poorer image quality of various functional imaging, the resulting dual-energy multiparameter imaging is a wider application than MRI. The multiple quantitative parameters from DECT exhibited potential in distinguishing T1-2 from T3-4 tumors. The NIC can reveal an increase in tumor neovascularization, it has been shown that iodine quantification in DECT correlates well with perfusion parameters [18, 28]. Our study showed the NIC values were higher in T1-2 carcinoma than in T3-4. We suspect that the difference in the NIC between T1-2 and T3-4 is due to the abundant blood vessels in the submucosal layer, so the T1-2 has higher NIC values. Besides, the Rho is the number of electrons per unit volume [29] and showed a linear relationship with tissue density. Our study demonstrated that advanced T staging causes higher electronic density of tumor; nevertheless, it is opposed to the result of NIC, which reflects a property of tissue that is distinct from contrast enhancement. Histologically, locally advanced tumor contained a high cellular density, so, we attribute the higher Rho in T3-4 to this potential increased connective tissue content.

There are several limitations to our study. First, our study was retrospective and the sample size was small. Further prospective studies with large numbers of patients are needed to be performed to verify our results. Second, we did not assess the status of lymph node. Because the exact pathologic correlation of surgically removed lymph nodes to the location of images was hard to complete match, preoperative lymph node staging is a worthy study. We encourage subsequent studies addressing this question. Third, we didn't compare the results of DECT with magnetic resonance imaging.

## Conclusions

This study showed that DECT has great advantages in evaluating T staging in patients with EC. The venous phase MEI (+)40 keV can improve the accuracy of evaluating T staging, and quantitative parameters derived from DECT also can help to identify T1-2 from T3-4.

## Abbreviations

CNR: Contrast-to-noise ratio
CT: Computer tomography
DECT: Dual-energy computed tomography
EC: Esophageal cancer
EUS: Endoscopic ultrasound
ID: Iodine density
MEI (+): Noise-optimized virtual monoenergetic images
NAC: Neoadjuvant chemotherapy
NCCN: National Comprehensive Cancer Network
NIC: Normalized iodine concentration
NPV: Negative predictive value
PEI: Polyenergetic images
PPV: Positive predictive value
Rho: Electron density
ROC: Receiver operating characteristic
ROI: Region of interest
SD: Standard deviation
SNR: Signal-to-noise ratio
UICC/AJCC: International Union Against Cancer/American Joint Committee on Cancer
Z_eff_: Effective atomic number
